# Identification of Driver Mutations and Risk Stratification in Lung Adenocarcinoma via Liquid Biopsy

**DOI:** 10.3390/cancers17081340

**Published:** 2025-04-16

**Authors:** Gopal P. Pathak, Rashmi Shah, Tony Reiman, Alison Wallace, Michael D. Carter, Stephanie Snow, John Fris, Zhaolin Xu

**Affiliations:** 1 Department of Pathology, QEII Health Sciences Centre, Dalhousie University, Halifax, NS B3H 1V8, Canada; gopal.pathak@dal.ca (G.P.P.); rashmi.shah@nshealth.ca (R.S.); michaeld.carter@nshealth.ca (M.D.C.); john.fris@nshealth.ca (J.F.); 2 Department of Oncology, Saint John Regional Hospital, Saint John, NB E2L 4L2, Canada; reimana@unb.ca; 3 Department of Biological Sciences, University of New Brunswick, Saint John, NB E2L 4L2, Canada; 4 Department of Medicine, Dalhousie University, Saint John, NB E2L 4L2, Canada; 5 Division of Thoracic Surgery, QEII Health Sciences Centre, Dalhousie University, Halifax, NS B3H 2Y9, Canada; alison.wallace@nshealth.ca; 6 Division of Medical Oncology, QEII Health Sciences Centre, Dalhousie University, Halifax, NS B3H 2Y9, Canada; stephanie.snow@nshealth.ca

**Keywords:** lung cancer, liquid biopsy, cfDNA, driver mutations, actionable mutations

## Abstract

Background: Liquid biopsy using plasma cfDNA has been established as a tool for informing the management of advanced-stage NSCLC. However, its effectiveness in early lung cancer detection, including the identification of high-risk cases, remains to be determined. Methods: We analyzed plasma cfDNA and matched tumors from 117 stage I–IV lung adenocarcinoma cases and compared the variants identified across all stages using the Oncomine Precision Assay on the Genexus^TM^ next-generation sequencing platform. Results: Cancer-specific mutations were detected in plasma from approximately 72% (84/117) of cases (all stages), with detection rates increasing by stage. Concordance between cfDNA and tumor tissue also increased with stage 0% (stage I), 19% (stage II), 45% (stage III), and 75% (stage IV). *KRAS* mutations were concordant in approximately 22% (6/27) of stage II and 46% (11/24) of stage III cases. Clinically important *EGFR* variants showed concordance in 11% (1/9) of stage II and 80% (8/10) in stage III/IV cases. Actionable mutations, targetable with FDA-approved drugs, were detected in 11% (4/37) of stage II, 27% (12/45) of stage III, and 55% (4/9) of stage IV cases, underscoring the potential of liquid biopsy for early detection of therapeutic targets. Moreover, co-occurring mutations with varying actionability were identified more frequently in plasma than in tumor tissues. Plasma detection of clinically important *KRAS* and *EGFR* variants was mostly associated with advanced-stage disease, suggesting the presence of these variants in plasma as a potential indication of disease progression. Conclusions: Liquid biopsy holds promise for identifying high-risk lung adenocarcinoma cases and serves as a complementary diagnostic tool in advanced stages, enhancing disease management strategies.

## Simple Summary

Liquid biopsy using plasma cell-free DNA (cfDNA) is a promising tool for managing advanced-stage non-small cell lung cancer (NSCLC), but its role in early cancer detection and risk stratification remains unclear. We analyzed plasma cfDNA and matched tumor tissues from individuals with various stages of lung adenocarcinoma to investigate the potential of liquid biopsy to detect cancer-specific mutations and assess concordance of detection. Liquid biopsy could detect cancer-specific mutations across all stages, with detection rates improving in advanced stages. Plasma detection of clinically significant *KRAS* and *EGFR* mutations was mainly associated with advanced-stage disease. Liquid biopsy detected actionable mutations in plasma, mostly from advanced-stage patients, highlighting the potential of the approach in identifying therapeutic targets for lung cancer. These findings demonstrate the importance of liquid biopsy in identifying high-risk cases and complementing the diagnosis of advanced-stage cases, thereby facilitating early therapeutic intervention.

## 1. Introduction

Non-small cell lung cancer (NSCLC) accounts for nearly 85% of all lung cancer cases, with an estimated 5-year survival rate of approximately 26.4% overall and around 5.8% for those diagnosed at stage IV, according to a 2021 study conducted in the USA [1]. In Canada, lung cancer is the leading cause of cancer-related death, with an estimated 5-year net survival rate of about 22% [2]. Detection of lung cancers at an early stage is crucial for patients to have the best chance of being treated for a cure and to maximize quality of life by potentially minimizing the intensity of required treatments [3]. Success of treatment is highest in early-stage but limited in advanced stages which continue to be associated with poor survival outcomes [4,5]. Unfortunately, many cases are identified at advanced stages due to the limitations of convenient and efficient early screening technologies [6,7].

Lung adenocarcinoma is the most common subtype of NSCLC and often harbours oncogenic driver mutations affecting key signaling pathways [1,4,8,9]. Tissue biopsy, though invaluable for obtaining detailed pathological and molecular insights into lung adenocarcinoma, presents several challenges. The invasiveness of the procedure, the challenges of accessing tumors in certain locations, and the time needed for diagnosis can contribute to significant delays in initiating treatment. Moreover, the presence of unidentified tumors or non-representative tissue samples may result in an incomplete understanding of the cancer’s molecular landscape and pathological status [10,11]. For the effective management of lung cancer, longitudinal monitoring of patients is crucial, which can involve serial imaging and tissue re-biopsy procedures for confirmation [12]. However, repeated interventions can be challenging due to procedural complexity and patient health considerations. Importantly, tumor development is a dynamic process, with the tumor’s genetic profile evolving to adapt to its changing environment and response to treatment exposure, leading to molecular changes over time [13,14,15]. Consequently, this change in the tumor molecular profile can lead to discrepancies between initial biopsy findings and later disease status, adding further complexity to effective therapeutic management.

In recent years, there has been growing interest in the application of liquid biopsy to identify cancer-specific actionable mutations from circulating cell-free tumor nucleic acids [16]. By detecting cancer-specific mutations through circulating tumor DNA/RNA (ctDNA/RNA) in blood samples, liquid biopsy can expedite diagnosis, particularly for patients with advanced-stage disease, and reduce the time to treatment initiation [17,18]. Most importantly, liquid biopsy offers a minimally invasive and real-time method to monitor tumor dynamics [19]. Moreover, it has the potential to detect ctDNA/RNA released by small primary tumors or micro-metastases, which may not be detectable using traditional imaging or tissue biopsy techniques [20].

Currently, liquid biopsy is predominantly used in advanced-stage NSCLC to identify molecular targets at the initial clinical diagnosis and to detect resistance mutations after progression on targeted therapies, particularly those targeting *EGFR* [21]. Additionally, it demonstrates the potential for monitoring treatment response, detecting therapy resistance, and tracking disease progression [21,22]. The technology has also been recommended for molecular profiling of advanced lung cancer patients to guide front-line treatment decisions when tissue biopsy is not feasible or accessible [23]. The non-invasive and repeatable nature of liquid biopsy makes it applicable for monitoring early-stage cases where regular surveillance is important to monitor treatment response and for detecting recurrence or metastasis. Furthermore, it holds the potential to address inequities in cancer diagnosis, particularly for underserved populations in rural or remote areas, as well as lower socioeconomic and marginalized groups, while also pioneering more accessible, personalized care models. For instance, peripheral blood can be drawn locally from the patients, which can prevent the barriers of cost and time as well as loss of income associated with traveling to major hospitals. These barriers disproportionately affect lung cancer outcomes, particularly for patients from socially disadvantaged and marginalized groups. Currently, lung cancer screening relies on radiation-based imaging technologies, often associated with prolonged wait and follow-up times, high false positive rates, and patient distress [24,25]. According to the American Lung Association (2022), only 5.8% of eligible Americans were screened for lung cancer in 2021, indicating a need for a convenient and accessible screening approach, among others [26]. Liquid biopsy holds promise as a potential screening modality, but limited data have not yet clearly defined the logistics and efficacy required to support its application for screening early-stage lung cancer [20]. Additionally, the potential of liquid biopsy for detecting early-stage cases, identifying risk groups, and longitudinal monitoring of early-stage cases remains largely unexplored [19].

To address these challenges, it is essential to conduct detailed studies that evaluate the stage-based effectiveness of liquid biopsy for detecting cancer-specific mutations [27]. In this study, we investigate potentially cancer-driving gene variant profiles in lung adenocarcinoma patients using plasma cell-free DNA (cfDNA) and matching tumor tissues across all four stages. We compare the results from plasma and matched tumor tissues to determine stage-specific driver mutation detection and concordance. Our findings highlight the effectiveness of liquid biopsy in advanced lung adenocarcinoma and its potential as a complementary tool for early detection, risk stratification, and monitoring across all stages of the disease.

## 2. Materials and Methods

### 2.1. Participant and Clinicopathological Data

Prospective lung cancer patients scheduled for tissue biopsy or surgical resection at the Queen Elizabeth II Health Sciences Centre in Halifax, Canada, were enrolled in this study. Enrolled patients had not received any prior therapy, such as immunotherapy, targeted therapy, chemotherapy, or radiotherapy, before the blood draw for the indicated lung cancer. All prospective patients underwent routine standard diagnostic and staging procedures, and confirmation of lung adenocarcinoma was achieved through pathological analysis of tissue samples. Patient demographic, clinical, pathology, and imaging data were collected from participant questionnaires and medical records. Molecular reports from clinical data were used as a reference to select potential cases for the study. We included cases with major lung cancer-specific mutations, such as *KRAS* and *EGFR*, along with a few other less common variants and those without clinically identified molecular variants. For the cancer-negative cohort, we included seven pathologically confirmed non-cancer cases, which consisted of lung fibrosis (*n* = 3), emphysema (*n* = 1), fibroelastosis (*n* = 1), hyperplasia (*n* = 1), and necrotizing granuloma (*n* = 1). None of these cases were found to have cancer for at least one year after the blood draw. All participants provided written informed consent. The study was approved by the Nova Scotia Health Authority’s Research Ethics Board (REB files #1013243 and #1011635).

### 2.2. Sample Collection and Processing

For subjects undergoing resection, blood samples were collected in EDTA tubes preoperatively on the same day as the surgical procedure and processed for plasma separation within 2–3 h of collection. Matching primary tumor tissues were collected from surgical samples immediately after surgery. For all resected cases, tumor size was determined from pathological specimens. The tissues were obtained as either fresh-frozen or formalin-fixed, paraffin-embedded (FFPE) samples. For five non-resected stage IV cases, matching tumor tissues were obtained from biopsy samples, and tumor size was assessed using imaging data. For three out of five non-resected stage IV cases, blood samples were acquired on the same day as the biopsy procedure. For the remaining non-resected stage IV cases, blood samples were acquired either a month before the biopsy (*n* = 1) or a month after the biopsy (*n* = 1). Plasma separated from the blood was stored at −80 °C until further processing. Total nucleic acid (TNA), comprising both DNA and RNA, was extracted from both plasma and tissue samples. We performed non-selective binding, washing, and clean-up of TNA bound to magnetic beads using the Maxwell^®^ RSC miRNA Plasma and Serum Kit (Promega, Madison, WI, USA), following the manufacturer’s protocol for Isolating Total RNA from Large Volumes of Plasma Using the Maxwell^®^ RSC miRNA Plasma and Serum Kit while avoiding nuclease treatment. In brief, about 3.2 mL of plasma (on average) was treated with proteinase K in the presence of a binding buffer for 15 min at 37 °C. The solution with magnetic beads from the kit was added to the proteinase K-treated plasma mix in the presence of isopropanol and incubated on a shaker at room temperature for 45 min. Subsequently, the mixture was centrifuged at 1000 rcf for 2 min at 4 °C to collect the magnetic beads, which were then resuspended in the sample buffer from well 1 of the Maxwell^®^ cartridges and washed and eluted to collect TNA using an automated Maxwell^®^ bench-top processor (Promega, Madison, WI, USA). Tumor tissues were extracted using Maxwell^®^ RSC simplyRNA Tissue or Maxwell^®^ RSC RNA FFPE Kit (Promega, Madison, WI, USA) without nuclease treatment to isolate TNA. The Maxwell^®^ RNA extraction kits employ magnetic beads that bind nucleic acids indiscriminately, which we repurposed for TNA extraction in this study. DNA and RNA in each sample were quantified with QuantiFluor^®^ RNA Systems (Promega, Madison, WI, USA) and QuantiFluor^®^ ONE dsDNA Dye (Promega, Madison, WI, USA) using the Quantus^TM^ Fluorometer (Promega, Madison, WI, USA).

### 2.3. Sequencing and Analysis

Next-generation sequencing analysis was performed on plasma and matched tumor tissues for all enrolled cases. The Ion Torrent^TM^ Genexus^TM^ integrated sequencer with the Oncomine Precision Assay (OPA) panel (Thermo Fisher Scientific, Waltham, MA, USA) was used for sequencing nucleic acids from plasma and tumor tissues. Plasma-based sequencing of DNA and RNA was performed using the same total nucleic acid input, following the manufacturer’s protocol. cfDNA was used as an input standard as our study focused on identifying tumor-specific SNV (single nucleotide variant) and indel (insertion and deletion) mutations in plasma. The cfDNA input ranged from 5 ng to 100 ng, based on availability, with a median of 21 ng. For solid tumors, sequencing of DNA and RNA was performed separately. The input amount for DNA or RNA from solid tumors was 17 ng for each case. The OPA panel allows the detection of the most prevalent and lung cancer-relevant variants across 50 genes, including potentially resistance-driving variants [28]. The panel can detect 2137 cancer-related indels and SNV hotspot mutations, 981 fusions, and 14 CNVs (copy number variation). The Genexus^TM^ Integrated Sequencer performed library preparation, sequencing, analysis, and reporting in an automated sample-to-result workflow. Integrated Genexus^TM^ software 6.8 was used for sequence alignment and variant calling. Reference Standards HD778 and HD779 (Horizon Discovery, Cambridge, UK) were used as cfDNA controls to evaluate the detection of standard variants. Oncomine Variants (6.8) filter chain was applied as a threshold to detect the hotspot variants. OncoKB^TM^ Human Genetic Variant Database (www.oncokb.org, accessed on 3 December 2024) was used to classify the actionable variants, which are annotated as Level 1 (FDA approved), Level 2 (standard of care), Level 3 (emerging evidence), and Level 4 (biological evidence) [29,30].

### 2.4. Data Visualization and Statistical Analysis

GraphPad Prism 8 for Windows (GraphPad Software, Boston, MA, USA), R (Version 4.4.3, The R Foundation, Vienna, Austria), and Microsoft Excel were used for data analysis and visualization. Categorical variables were evaluated by Fisher’s exact test and continuous variables were evaluated by student t-test. All hypothesis tests were two-sided, and a *p* value of less than 0.05 was considered statistically significant.

## 3. Results

### 3.1. Participants and Sample Overview

**Figure 1:**
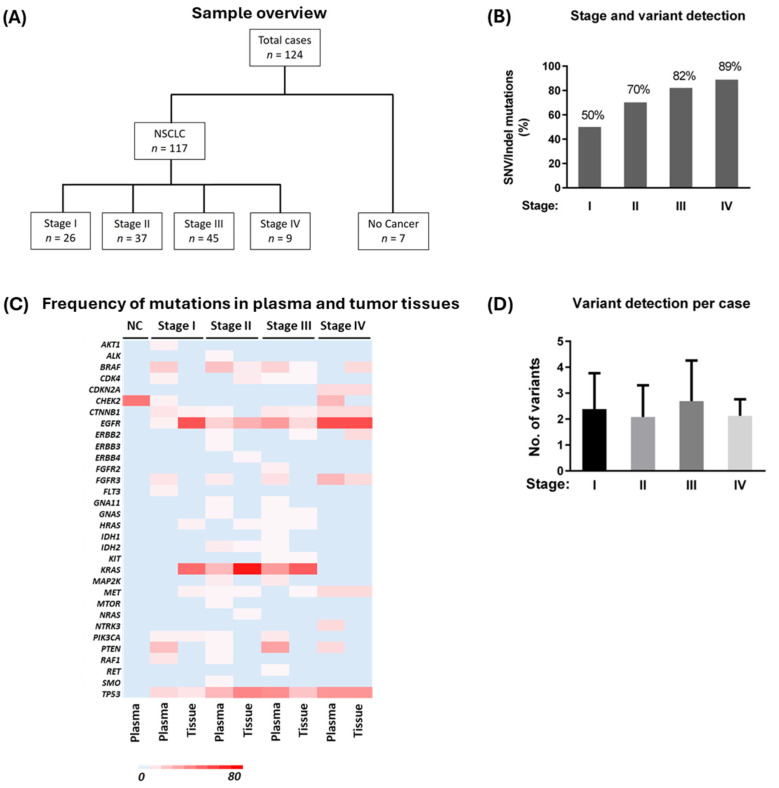
(**A**) Synopsis of lung adenocarcinoma and cancer-negative cases used in this study. (**B**) Frequency of SNV/indel variants detected from cfDNA of lung adenocarcinoma patients. NC denotes non-cancer cases. (**C**) Heatmap showing the percentage of various hotspot mutations in plasma and tissue samples from all four stages of lung adenocarcinoma and cancer-negative cases. Only genes with detected variants are included. Proportion of mutations is calculated for each stage. (**D**) Average number of variants detected per patient from cfDNA. On average, 2.38 (SD = 1.39), 2.08 (SD = 1.22), 2.69 (SD = 1.59), and 2.12 (SD = 0.64) variants were identified in each stage I, II, III, and IV, respectively. The data are from positively detected cases only.

A total of 117 lung adenocarcinoma (including 2 pleiomorphic carcinoma with adenocarcinoma component) patients at different pathological stages and seven cancer-negative cases (as described in Materials and Methods, Section 2) were selected for this study (Figure 1A). The average primary tumor size was 4.1 cm (SD = 2.56) for all cases. Nine patients had evidence of distant metastasis. There were 68 female and 49 male patients in the lung adenocarcinoma-positive cohort, with a median age of 67 years (SD = 8). Clinical and demographic data are given in Table 1. The mean total cfDNA yield was higher (23.95 ng/mL, *n* = 117) in the lung adenocarcinoma cohort than in the non-cancer cohort (9.57 ng/mL, *n* = 7) (*p* = 0.081).

### 3.2. Variant Detection in Plasma cfDNA and Matched Tumor Tissues from Stage I to IV Patients

We detected up to two copies of reference SNV/indel variants from the cfDNA control set (Supplementary Tables S1 and S2). In the clinical samples, SNV/indel hotspot mutations were detected in cfDNA from 50% (13/26), 70% (26/37), 82% (37/45), and 89% (8/9) of stage I, II, III, and IV cases, respectively (Figure 1B and Table 2). SNV/indel hotspot mutations were present in the tumor tissues in 100% (26/26), 100% (37/37), 75% (34/45), and 89% (8/9) of stage I, II, III, and IV cases, respectively. Tissue-only detection of hotspot mutations was found in 50% of stage I, 30% of stage II, and 9% of the stage III cohort, whereas plasma-only detection of such mutations was found in 15% of the stage III cohort only (Table 2). Overall, we detected 197 SNV/indel hotspot mutations from plasma cfDNA across all 117 cancer cases (stages I to IV), compared to 154 such mutations from matching tumor tissues. We detected 6 indel variants from plasma, whereas 24 such variants were detected from tumor tissues. Plasma detected indel variants were *EFGR* Exon 18 (*n* = 1) or Exon 19 (*n* = 5) deletion. *ALK* fusions (*n* = 2), detected in tissue RNA from SNV/indel mutation-negative tissue samples, were not detected in corresponding cfDNA/RNA. Similarly, *CDKN2A* loss of function (*n* = 1) and *EGFR* copy number variation (*n* = 1), identified in tissue samples from stage III cases that tested negative for SNV/indel mutations, were not detected in plasma samples. The detection of driver mutations was more common in cfDNA from advanced-stage cases. *PTEN* mutations were found in cfDNA more frequently than in tumor tissues (Figure 1C). Interestingly, we did not find any variants other than *CHEK2* R523H (3/7 cases) in non-cancer cases. The *CHEK2* R523H variant in these cases is favored to be of germline origin or non-specific mutations and probably not related to underlying malignancy in non-cancer cases [31]. Mutations detected in individual oncogenes and their frequency in the cohort are given in Figure 1C. On average, about two variants were detected per patient among the liquid biopsy-positive cases in all four stages (Figure 1D).

### 3.3. Concordance of Detection of Variants Across All Four Stages of NSCLC

**Figure 2:**
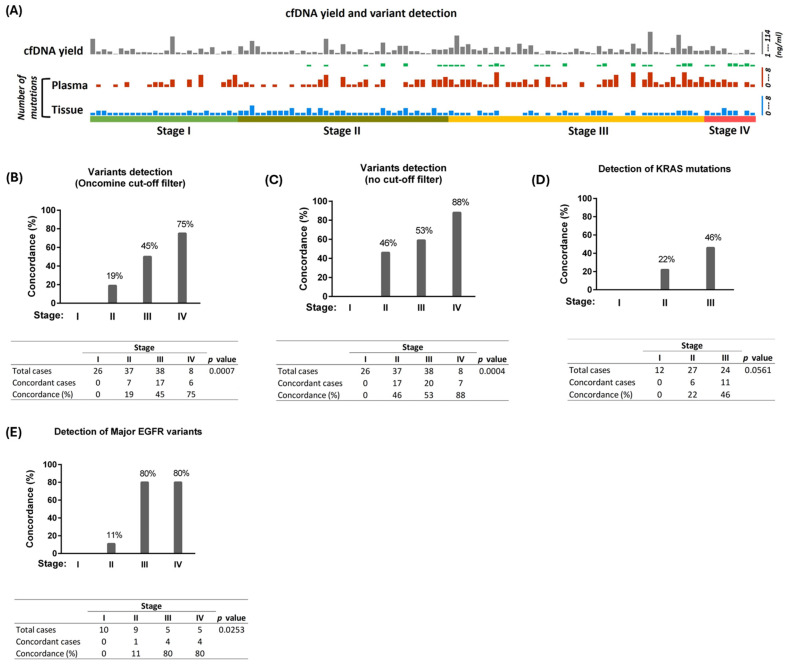
(**A**) Mutation detection from tissue and cfDNA in each lung adenocarcinoma case. Concentration of cfDNA (per mL of plasma) and the number of mutations detected in each case are shown. Green boxes denote the concordant (cfDNA-tissue) cases, with thick boxes representing two concordant mutations. (**B**) Concordance of detection of SNV/indel variants at given stages of lung adenocarcinoma (with Oncomine cutoff filter). (**C**) Concordance of detection of SNV/indel variants at given stages of lung adenocarcinoma (without Oncomine cutoff filter). For stage III, variant-positive tumor tissues (*n* = 38) are used as reference for the analysis in (**B**,**C**). Negative concordances (two from stage III and one from stage IV) are not included in the analysis. (**D**,**E**) Concordance of detection of clinically significant *KRAS* mutations (**D**) and *EGFR* variants (**E**) at specific stages. Fisher’s exact test was used for statistical analysis.

The frequency of variant detection and concordance between plasma and tumor tissues for each case is presented in Figure 2A. Concordance between matched tumor tissue and plasma was not found in any of the 26 cases from the stage I cohort. In the stage II cohort, concordance of detection was found in 9 events from 7/37 (19%) cases (Figure 2A,B). The concordance in stage II was found for mutations in *EGFR*, *KRAS*, *MET*, and TP53 genes. Among stage III cases, plasma cfDNA showed concordance in 23 events from 17/38 (45%) cases (Figure 2B). Additionally, two cases did not have any variants detected in plasma or matched tumor tissues (negative concordance). The genes with concordant variants found in stage III were *CTNNB1*, *EGFR*, *KIT*, *KRAS*, and *TP53*. Both *CTNNB1* (2/2) and *KIT* (1/1) variants showed 100% concordance in stage III, whereas *TP53* had 50% (4/8) concordance. In stage IV, positive concordance was found in 6/8 cases (75%), and one case had negative concordance. Moreover, 100% concordance was found in *CTNNB1* (1/1), *CDKN2A* (1/1), *FGFR3* (1/1), and *MET* Exon 14 skipping (1/1) in the stage IV cohort. Next, we analyzed the unfiltered sequence data (no filter chain applied) to search for the tumor-specific driver mutations in cfDNA that were not called by the bioinformatic pipeline with the Oncomine filter applied. The concordance of detection at this parameter was 0/26 (0%), 17/37 (46%), 20/38 (53%), and 7/8 (87%) in stages I, II, III, and IV, respectively (Figure 2C). Although manual identification of variants from unfiltered data increased the concordance, this approach is only possible in the presence of tumor sequencing data. For all other analyses in the manuscript, we used Oncomine Variants (6.8) filter chain cutoff parameters.

### 3.4. Detection of Major Driver Mutations

Next, we assessed the efficacy of liquid biopsy in detecting clinically significant *KRAS* and *EGFR* variants, which are the primary NSCLC driver genes in our study cohort. We analyzed cfDNA and tissue DNA to determine the sensitivity of variant detection for these important driver genes from selected lung adenocarcinoma cases. *KRAS* mutations were present in tumor tissues from 12 (46%) stage I, 27 (73%) stage II, and 24 (53%) stage III cases in the study cohort. Similarly, clinically important *EGFR* variants were present in tumor tissues from 14 (54%) stage I, 9 (24%) stage II, 5 (11%) stage III, and 5 (55%) stage IV cases in the cohort. Among them, *EGFR* Exon 19 deletions (*n* = 21) and L858R mutations (*n* = 10) (including one case with both Exon 19 deletion and L858R) were the most common clinically significant *EGFR* variants present in the cohort. None of the *KRAS* or *EGFR* variants identified in stage I tumors were detected in plasma. The concordance of detection between cfDNA and tissue DNA for *KRAS* mutations was 22% (6/27) in stage II and 46% (11/24) in stage III (Figure 2D). None of the stage IV cases available in our cohort had *KRAS* mutations identified from tissue or plasma analysis. Concordance of detection for clinically important *EGFR* variants between cfDNA and tumor DNA in stage II cases was 11% (1/9 cases) and 80% in both stage III (4/5) and stage IV (4/5) cases (Figure 2E). These data demonstrate that clinically important lung cancer driver mutations could be detected by liquid biopsy in most stage III and IV cases. Importantly, all *EGFR* indel variants detected from plasma (*n* = 6, from stage II-IV) were concordant with the matching tumor tissues. *TP53* mutation, along with *EGFR* Exon 19 deletion, was identified in the cfDNA of one case in both stage II (concordant for both *EGFR* Exon 19 deletion and *TP53* mutation) and stage III (concordant for *EGFR* Exon 19 deletion only) cohorts. Furthermore, a combination of *TP53* and *KRAS* G12X was identified in cfDNA from one stage II case (concordant for both *TP53* and *KRAS* mutations) and six stage III cases (four cases concordant for both *TP53* and *KRAS* mutations and two cases concordant for *KRAS* mutation only).

### 3.5. Presence of Actionable Mutations

**Figure 3:**
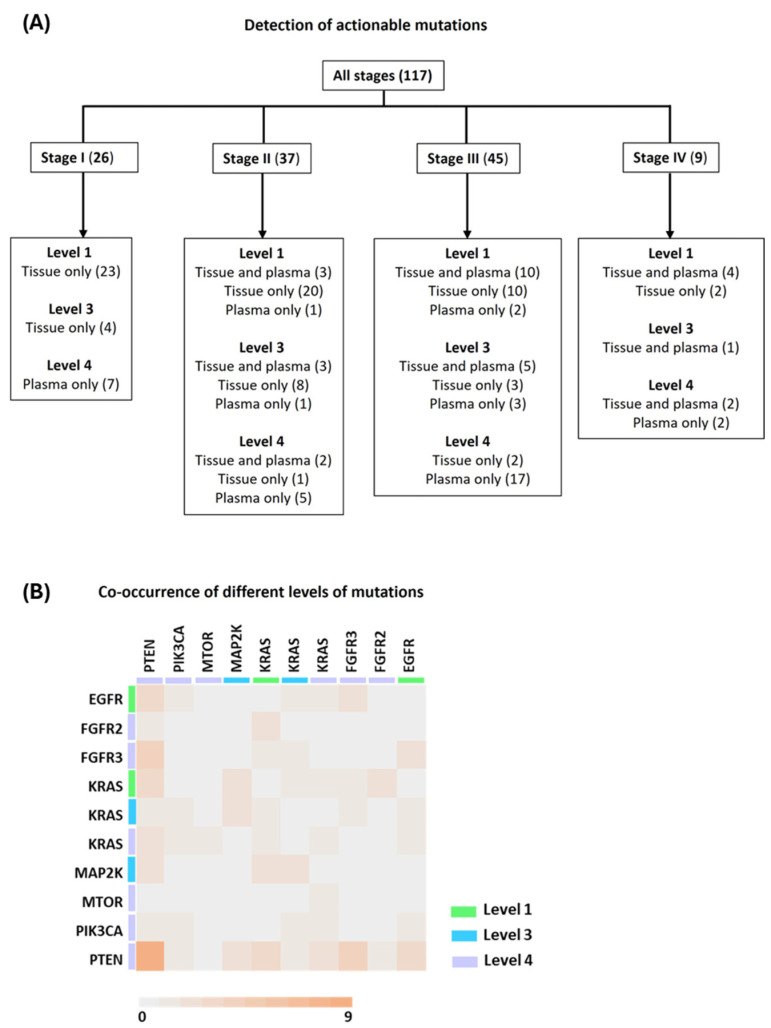
(**A**) Detection of various levels of actionable variants from plasma and matched tumor tissues. Number of cases are given in parentheses. (**B**) Heatmap showing the co-occurrence of various levels of mutations as defined by OncoKB^TM^ in plasma from each case. Scale shows the number of cases and color bars represent various levels of biomarkers as defined.

We analyzed actionable mutations in plasma cfDNA using OncoKB^TM^ as a reference. Level 1 actionable mutations, for which FDA-approved targetable drugs are available, were found in plasma from 11% (4/37) of stage II, 27% (12/45) of stage III, and 44% (4/9) of stage IV cases (Figure 3A). None of the stage I cases had Level 1 mutations detected in plasma. No Level 2 specific mutations were detected in plasma or tumor tissue samples from any stage. Level 3 mutations were detected in plasma from 0/26 (0%), 4/37 (11%), 8/45 (18%), and 1/9 (11%) stage I, II, III, and IV cases, respectively. Level 4 mutations were detected in 7/26 (27%), 7/37 (19%), 17/45 (38%), and 4/9 (44%) stage I, II, III, and IV cases, respectively. One stage II (*ERBB2* R868W) and two stage III (*KRAS* G12C and *EGFR* T790M) cases presented plasma-only detection of Level 1 actionable mutations (Figure 3A). The results demonstrate that the detection of actionable mutations in plasma cfDNA increases with disease stage (Supplementary Table S3). Interestingly, co-occurring mutations were detected in plasma samples from multiple cases involving Level 1, 3, and 4 variants (Figure 3B). A total of 39 distinct gene combinations with varying mutation levels (Level 1, 3, and 4) were detected in cfDNA from 17 cases across various stages: 1 in stage I (1 case), 4 in stage II (4 cases), 32 in stage III (10 cases), and 2 in stage IV (2 cases) (Supplementary Table S4). In contrast, only 4 similar combinations were found in tumor tissues from 4 cases (1 in stage I, 1 in stage II, and 2 in stage IV). None of the specimens contained two or more co-occurring Level 1 actionable mutations in cfDNA.

## 4. Discussion

We analyzed hotspot driver gene variants in plasma and matched tumor tissues from cases across all four stages of lung adenocarcinoma. Interestingly, we identified several hotspot driver mutations in cfDNA, even in early-stage cases, suggesting that cfDNA might serve as a non-invasive indicator of tumor presence. Concordance in the detection of clinically important mutations in cfDNA varied by stage; no tumor-specific mutations were detected in cfDNA of stage I cases, but detection rates increased with stage thereafter. While this most likely reflects a correlation between tumor burden and DNA shedding, tumor heterogeneity could also play a role. Higher genetic heterogeneity has been reported in early-stage disease, with selective survival of tumor cells containing key driver mutations (such as *KRAS*, *EGFR* variants, etc.) that impart enhanced invasive potentials [32,33,34]. Consequently, such aggressive driver variants, which may confer a fitness advantage over other mutations, may continue to survive and promote tumor progression; in early-stage disease, these variants may only be present in a small proportion of tumor cells, potentially remaining undetected in cfDNA [35,36]. Further studies are needed to explore the relationship between molecular profiles and tumor evolutions in early-stage lung cancers.

We observed high concordance between tissue and plasma in stage III and IV cases but found no concordance in stage I cases and only minimal concordance in stage II. This is consistent with other studies, which reported over 65% concordance in mutation detection between cfDNA and tumor tissue in advanced-stage lung cancers [37,38,39,40]. Such agreement is found particularly for major actionable driver mutations, including *EGFR* and *KRAS* [37,41,42,43]. Detecting mutations through liquid biopsy in early-stage (I and II) cancer remains challenging, which may be due to very low levels of circulating tumor DNA in these cases, assay limitations, or a combination of factors [44,45]. Reflexive tissue testing is recommended when a liquid biopsy fails to identify a driver mutation [46]. The detection rate can be increased when a less rigorous filtering threshold is applied, but such an approach can potentially contribute to false positive detection, especially when no reference tumor tissue is available for comparison. On the other hand, more stringent filtering may lead to higher false negative results. To improve detection rates and reduce both false negatives and positives, it is crucial to expand the range of oncogene markers in sequencing panels, develop advanced algorithms to analyze molecular changes in cfDNA and increase assay sensitivity.

Our data highlight the potential of liquid biopsy to identify clinically important driver mutations, especially when timely tumor tissue analysis is unfeasible or when tissue biopsies miss key mutations due to non-representative sampling. Since plasma detection of clinically important *KRAS* and Level 1 *EGFR* variants (such as Exon 19 deletions, L858R, and T790M) is primarily associated with advanced stages, finding these mutations in cfDNA from tissue biopsy-classified early-stage cases could signal that distant spread may have already occurred, with micro-metastatic disease undetectable by radiographic staging. Such information can be valuable for determining cases needing further monitoring and assessment or even escalation of systemic therapy delivered in the curative-intent setting. *KRAS* is the most prevalent driver mutation in NSCLC, with *EGFR* being the next most common [4,47,48,49]. Importantly, actionable *KRAS* and *EGFR* alterations make up a significant portion of overall actionable alterations in advanced NSCLC (adenocarcinoma), together accounting for 44% in Western populations and up to 60% in Asian populations [49]. Our results show a higher concordance in detecting these mutations among stage III/IV lung adenocarcinoma patients, supporting the utility of liquid biopsy as a rapid, complementary diagnostic tool for identifying key mutations in lung cancers and enabling timely therapeutic intervention. Although tissue-based analysis is one of the most reliable approaches to date to study the mutation profiles, tumor tissue analysis alone may fail to detect certain actionable or resistance mutations, potentially limiting targeted treatment options [37,39,43,50]. For instance, we detected key actionable mutations, such as *KRAS* G12C and *EGFR* T790M, exclusively in plasma in certain stage III cases, underscoring the importance of combining liquid biopsy with tissue biopsy for more comprehensive lung cancer management. Similar findings have been reported in stage IV cases, where actionable variants were identified exclusively through ctDNA, further supporting the combined use of tissue and cfDNA analysis to improve mutation detection [43]. It is worth noting that *EGFR* T790M, primarily associated with acquired resistance in lung cancer after *EGFR* tyrosine kinase inhibitors therapy, can also be found in a small number of treatment-naive cases [51]. We observed the plasma detection of *PTEN* mutations from the cases at all stages. *PTEN* variants are infrequently observed in NSCLC, and their clinical significance remains unclear due to their low prevalence and the limited availability of data [52]. While no cases with two or more co-occurring Level 1 actionable mutations in cfDNA were identified, liquid biopsy detected several co-occurring mutations more frequently than tumor testing. This highlights the diversity in oncogene activation achieved through tumor clonal evolution [14,53]. Additionally, it underscores the utility of cfDNA, relative to tissue testing, in capturing this heterogeneity and potentially improving patient management. Level 4 mutations were detected more frequently in plasma cfDNA, suggesting an expanded role for liquid biopsy as new targeted therapies continue to emerge.

This study aimed to identify the effectiveness of liquid biopsy in detecting major NSCLC variants at different stages using selected patient samples in clinical settings. In our cohort, most cases contained cancer-specific SNV/indel mutations, primarily *KRAS* and *EGFR* variants with significant clinical importance, with fewer instances of other variants. Further studies with a larger cohort are needed to evaluate the concordance of less-represented variants in our study, such as fusions and CNVs. Tracking tumor biomarkers in plasma offers a convenient approach for monitoring disease progression over time. However, many biomarkers are not specific to a single cancer type, and their levels are often low in early-stage tumors, making exclusive reliance on liquid biopsy for diagnosis challenging.

In addition to NGS-based analysis, non-genetic methods, such as liquid biopsy metabolomics, can provide additional benefits for early detection and treatment response monitoring [54]. Unlike NGS-based techniques that focus on detecting tumor mutations, metabolomic profiling can identify small-molecule metabolites that indicate changes in tumor-related metabolism and biochemical pathways [55]. Integrating metabolomic and genetic liquid biopsy data may improve detection accuracy in NSCLC, providing a comprehensive approach to precision oncology. Digital droplet PCR is a popular method for targeted mutation analysis in cancers, which has high specificity but limited multiplexing ability and applications [56,57]. NGS panels, which encompass targeted and whole-genome sequencing, allow for more comprehensive variant profiling but may require adequate ctDNA/RNA fractions for the detection of driver variants [19]. A combination of data from tumor-specific methylation, tumor mutation burden, and tumor factors can provide crucial information for enhancing the early detection of cancer via liquid biopsy [19].

The insights gained from liquid biopsy can be valuable for monitoring and further assessment and management of individual cases. The capacity of liquid biopsy to detect actionable variants and key driver mutations makes it a valuable tool for guiding personalized treatment strategies while also holding promise for future lung cancer screening in high-risk but asymptomatic individuals. Our findings emphasize the potential of liquid biopsy in risk stratification and demonstrate its clinical utility as a complementary diagnostic tool in lung cancers.

## 5. Conclusions

In conclusion, our study highlights the potential of liquid biopsy as a non-invasive tool for detecting driver mutations in lung adenocarcinoma, particularly in advanced stages where concordance with tumor tissue is high. While early-stage cancer detection using liquid biopsy remains challenging, it can complement tissue testing by identifying actionable mutations like *KRAS* and *EGFR* even in non-metastatic and locally advanced stages, guiding personalized treatment in a timely manner. Its ability to detect resistance mutations and molecular signatures that may reflect tumor heterogeneity further enhances its utility in cancer management. However, sensitivity and specificity limitations in early-stage disease call for further research to optimize assays, develop robust algorithms, and expand biomarker panels. Overall, liquid biopsy holds promise for identifying high-risk cases, monitoring progression, and guiding therapy, underscoring its role as a transformative tool in lung cancer care.

## Figures and Tables

**Table 1:** Patient characteristics.

Characteristics	Stage I	Stage II	Stage III	Stage IV
Total cases (*n* = 117)				
No. of cases at each stage	26	37	45	9
Average primary tumor size (cm)	2.55	3.42	5.66	3.55
Median age (SD)	69 (8)	67 (7)	66 (8)	67 (7)
Sex				
Female	18 (69%)	25 (68%)	21 (47%)	4 (44%)
Male	8 (31%)	12 (32%)	24 (53%)	5 (56%)

**Table 2:** Variant detection from plasma and tumor samples.

Characteristics	Number of Cases (%)			
SNV/Indel variants	Stage I	Stage II	Stage III	Stage IV
Plasma+, Tissue+	13 (50)	26 (70)	30 (67)	8 (89)
Plasma+, Tissue−	0 (0)	0 (0)	7 (15) *	0 (0)
Plasma−, Tissue+	13 (50)	11 (30)	4 (9)	0 (0)
Plasma−, Tissue−	0 (0)	0 (0)	4 (9) **	1 (11)
CNV/fusion/LoF	Stage I	Stage II	Stage III	Stage IV
Plasma+, Tissue+	0 (0)	0 (0)	0 (0)	0 (0)
Plasma+, Tissue−	0 (0)	0 (0)	0 (0)	0 (0)
Plasma−, Tissue+	0	0	4 (9)	0 (0)
Plasma−, Tissue−	26 (100)	37 (100)	41 (91)	9 (100)

* Out of seven SNV/indel Plasma+, Tissue− cases, one has *CDKN2A* LoF and the other has *ALK* fusion detected from tissue only. ** Out of four SNV/indel Plasma−, Tissue− cases, one has *EGFR* CNV and the other has *ALK* fusion detected from tissue only. CNV: copy number variation, LoF: loss of function.

## Data Availability

Upon request, non-confidential information may be available from the corresponding author.

## Supplementary Materials

The following supporting information can be downloaded at: https://www.mdpi.com/article/10.3390/cancers17081340/s1; Supplementary Table S1. Detection of variants from positive control (with 20 ng input from 1% stock-HD778). Expected and detected allele frequency (AF) are presented (*n* = 2); Supplementary Table S2. Detection of variants from positive control (with 20 ng input from 0.1% stock-HD779). Expected and detected allele frequency (AF) are presented (*n* = 3); Supplementary Table S3. Identification of levels of mutations in cfDNA based on the OncoKB database; Supplementary Table S4. Combinations of unique variants from different actionable levels, as categorized in OncoKB, present in cfDNA of various lung adenocarcinoma cases across different stages. A gene with multiple variants at the same level is considered only once if present multiple times within the same case.

## Abbreviations

The following abbreviations are used in this manuscript.

cfDNA	Cell-free DNA
cfRNA	Cell-free RNA
CNV	Copy number variation
ctDNA	Circulating tumor DNA
FFPE	Formalin-fixed paraffin-embedded
Indel	Insertion and deletion
NSCLC	Non-small cell lung cancer
SD	Standard deviation
SNV	Single nucleotide variant
TNA	Total nucleic acid
