# Supplementary material for: Identification of Driver Mutations and Risk Stratification in Lung Adenocarcinoma via Liquid Biopsy
Source: Cancers (Basel). 2025 Apr 16;17(8):1340. doi: 10.3390/cancers17081340 (PMC12025768; doi:10.3390/cancers17081340)
Supplement: Supplementary file 1 [file cancers-17-01340-s001.zip › cancers-3524973-supplementary.pdf]

Supplementary Table S1. Detection of variants from positive control (with 20 ng input from 1% stock-HD778). Expected and detected allele frequency (AF) are presented (*n* = 2).

Gene	Amino Acid Change	Mutant (copies/rn)	Expected AF (%)	Detected AF 1 (%)	Detected AF 2 (%)	Average AF (%)
EGFR	V769_D770insASV	19	1.00	0.80	2.20	1.50
EGFR	E746_A750del	30	1.00	1.00	1.50	1.25
EGFR	L858R	40	1.00	0.50	0.00	0.25
EGFR	T790M	24	1.00	0.20	1.00	0.60
KRAS	G12D	36	1.30	1.20	1.00	1.10
NRAS	A59T	56	1.30	1.80	0.20	1.00
NRAS	Q61K	46	1.30	0.50	0.50	0.50
PIK3CA	E545K	44	1.30	1.50	1.20	1.35

Copies/rn: copies per reaction. Average data from two separate runs.

Supplementary Table S2. Detection of variants from positive control (with 20 ng input from 0.1% stock-HD779). Expected and detected allele frequency (AF) are presented (*n* = 3).

Gene	Amino Acid Change	Mutant (copies/rn)	Expected AF (%)	Detected AF 1 (%)	Detected AF 2 (%)	Detected AF 3 (%)	Average AF (%)
EGFR	V769_ D770insASV	2	0.10	0.40	0.10	0.10	0.20
EGFR	E746_A750del	2	0.10	0.00	0.20	0.00	0.07
EGFR	L858R	4	0.10	0.00	0.00	0.10	0.03
EGFR	T790M	3	0.10	0.30	0.10	0.20	0.20
KRAS	G12D	5	0.13	0.10	0.10	0.10	0.10
NRAS	A59T	5	0.13	0.10	0.10	0.10	0.10
NRAS	Q61K	5	0.13	0.00	0.02	0.10	0.04
PIK3CA	E545K	5	0.13	0.20	0.00	0.10	0.10

Copies/rn: copies per reaction. Average data from three separate runs.

Supplementary Table S3. Identification of levels of mutations in cfDNA based on the OncoKB database.

Category	No. of cases				*p* value
	Stage I	Stage II	Stage III	Stage IV	
Level 1	0	4	12	4	0.010
Level 3	0	4	8	1	0.177
Level 4	7	7	17	4	0.466
Total cases	26	37	45	9	

Supplementary Table S4. Combinations of unique variants from different actionable levels, as categorized in OncoKB, present in cfDNA of various lung adenocarcinoma cases across different stages. A gene with multiple variants at the same level is considered only once if present multiple times within the same case.

Combinations	Stage I	Stage II	Stage III	Stage IV	*p* value
	No. of events	No. of events	No. of events	No. of events	
Level1-Level1	0	0	0	0	-
Level1-Level3	0	0	4	0	0.190
Level1-Level4	0	1	12	2	0.004
Level3-Level3	0	1	1	0	1.0
Level3-Level4	0	1	5	0	0.253
Level4-Level4	1	1	10	0	0.044
All Levels	1	4	32	2	<0.0001
Total cases	26	37	45	9	
